# Structural Alerts for Aneuploidy Prediction: Are We There Yet?

**DOI:** 10.3390/toxics14050363

**Published:** 2026-04-24

**Authors:** Erika Maria Ricci, Cecilia Bossa, Francesca Marcon, Lorenza Troncarelli, Chiara Laura Battistelli

**Affiliations:** Environment and Health Department, Istituto Superiore di Sanità, 00161 Rome, Italyfrancesca.marcon@iss.it (F.M.); chiara.battistelli@iss.it (C.L.B.)

**Keywords:** structure-activity relationship models, structural alerts, toxicological dataset, genotoxicity, aneuploidy, risk assessment

## Abstract

Assessing genotoxicity, specifically gene mutations and chromosomal aberrations, is fundamental to chemical risk assessment. Notably, the early identification of an aneugenic mechanism is of crucial importance, allowing, in principle, for a threshold-based risk assessment approach. To investigate this issue while pushing towards innovation in risk assessment by leveraging New Approach Methodologies, in silico approaches stand out as a particularly promising avenue. Building on these premises and given the lack of QSAR models for aneuploidy in the public domain, the present study exploited the genotoxicity-relevant alert lists implemented in the OECD QSAR Toolbox to base the investigation of structure-activity relationships for aneuploidy. To address the lack of relevant structured data resources, a dataset of 65 confirmed aneugenic substances was specifically curated and designed for the study. The results highlighted widely differing performances among the various profilers, confirming a general limited discriminatory power for aneuploidy. On the other hand, a granular analysis of the results from individual structural alerts enabled the successful isolation of some features associated with the aneugenic mode of action. Moreover, a subset of tubulin-binding chemicals was investigated to determine whether targeting a specific protein improves the characterization of toxicological alerts. The findings provide a refined definition of specific toxicity determinants for tubulin binders and serve as a promising tool for early hazard assessment, potentially informing relevant AOPs. While the computational approach appears promising, the overarching challenge that emerges is the limited availability of well-curated experimental data. In fact, reliable data on aneuploidy are scarce and fragmented across the literature. Furthermore, existing compilations of micronucleus study results are often complicated by conflicting interpretations.

## 1. Introduction

Genotoxicity refers to toxic effects exerted on DNA, encompassing gene mutation, structural chromosomal aberrations (clastogenicity), and numerical chromosomal aberrations (aneugenicity and polyploidy) [1,2]. Assessment of genotoxic potential is a fundamental component of chemical risk assessment and serves as an important prescreening for predicting carcinogenic potential [3]. Since no single test can detect all genotoxic events, regulatory frameworks often mandate the use of “test batteries”, typically starting with in vitro tests and proceeding to in vivo confirmation tests when necessary [4,5]. Currently, genotoxicity assessment is mainly hazard-based, assuming that genotoxic compounds have the potential to damage DNA at any level of exposure [6]. However, aneugens, unlike DNA-reactive genotoxicants, are considered to be non-DNA-reactive, acting on the mitotic spindle or other components of the cell division machinery [7,8]. Therefore, an aneugen risk assessment may, in principle, follow a threshold-based approach, assuming that low doses may not elicit adverse effects due to redundancy (or multiplicity) in cell-division targets (or due to functional redundancies in the mitotic apparatus) [6].

While several specific mechanisms and molecular targets can lead to aneuploidy, certain mechanisms are considered more prevalent and have been more extensively studied, such as tubulin binding and Aurora kinase B inhibition. Tubulin binding acts as a molecular initiating event that disrupts microtubule dynamics, leading to spindle abnormalities and impaired chromosome attachment. Conversely, aurora kinase B inhibition primarily prevents cytokinesis, the physical division of the cell, leading to binucleation. These binucleated cells often develop multipolar spindles in subsequent divisions, which significantly increases the frequency of chromosome missegregation.

Regardless of the specific mechanism that gives rise to aneuploidy, the micronucleus (MN) test is the key assay for detecting both aneugens and clastogens. To differentiate between clastogenic and aneugenic events, the MN test can be combined with techniques such as kinetochore staining (CREST) or fluorescence in situ hybridization (FISH) with pan-centromeric probes. These methods help determine whether the micronucleus originated from a chromosome fragment (acentric) or a whole chromosome, making the test a powerful and versatile tool in genetic toxicology [9]. Currently, the in vivo micronucleus (OECD TG 474) is the preferred test for in vivo follow-up of substances with in vitro evidence of chromosomal aberrations [5].

In recent decades, the 3R (Reduction, Refinement, Replacement) principles have driven the development and adoption of alternative methodologies to animal experimentation [10]. Among these alternatives, non-testing methods leverage pre-existing data and information derived from molecular structure to predict biological or toxicological results. These methods include quantitative structure-activity relationship (QSAR) models, which provide numerical activity predictions (e.g., lethal dose 50%, LD50), and structure-activity relationship (SAR) models, which provide qualitative, binary results (e.g., toxic/non-toxic). SAR models rely on structural alerts (SAs), which codify a mechanistic process corresponding to a given toxicological effect.

Both the literature and the public domain offer several models for the prediction of the in vivo MN test outcome (e.g., [11,12,13]), while there is a recognized gap, as no publicly available SAR model currently exists that specifically predicts the potential aneugenicity of chemical substances. Notably, in its guidance on aneugenicity assessment [2], the European Food Safety Authority (EFSA) has recommended further investigation into possible structure-activity relationships for aneugens to improve their characterization and assessment. The absence of specific models for aneugenicity prediction is largely due to the lack of curated datasets of substances acting specifically via this mechanism. As a matter of fact, structured datasets of MN results (in vitro and/or in vivo) available in the public domain do not contain details on the mechanism of action (i.e., aneugenicity or clastogenicity). This could be attributed either to the fact that the tests were performed without applying methods for differentiation between clastogens and aneugens or to the omission of their results in the metadata of the databases. It must be noted that the application of FISH or CREST methods in an MN assay is reported as optional in OECD TGs 487 and 474.

The present study aims to investigate structure-activity relationships of confirmed aneugens through the application and potential refinement of selected existing models relevant for genotoxicity assessment. To this end, the first phase of the work focused on establishing a curated dataset of aneugenic substances. Subsequently, we screened established genotoxicity alert lists and relevant profilers for general mechanistic screening to determine whether any specific features, although embedded in profilers originally not designed to detect the aneugenicity endpoint, could prove relevant to aneuploidy analysis. To this aim, the predictive power of the selected existing genotoxicity-relevant models was evaluated on the dataset of aneugenic chemicals. These results were then compared with the performance obtained from the application of the same profilers to chemicals used as negative controls (i.e., clastogens and non-genotoxic chemicals). Moreover, an in-depth study of the most frequently identified SAs and functional groups was performed to highlight and refine structural determinants for aneugenesis.

The ultimate goal of this work is to improve the availability of scientific tools for hazard identification of this specific endpoint. By investigating structural determinants associated with aneugenicity, this study may contribute to the improvement of the applicability of mechanistically informed adverse outcome pathways (AOPs). The integration of structure-based insights into the AOP framework aligns with the goals of the New Approach Methodologies (NAMs), which promote the use of mechanistic, non-animal-based strategies, such as in silico modeling and pathway-based assessment, for predicting toxicological outcomes. By enhancing our ability to predict aneugenic potential through SAR analysis, this study supports the broader shift toward NAMs and the development of next-generation risk assessment tools.

**Figure 1:**
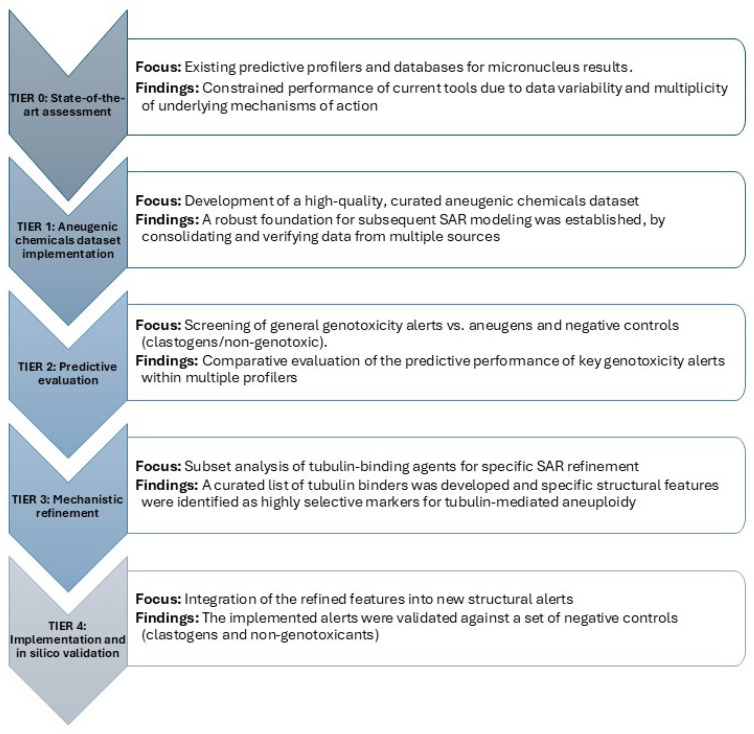
Overview of the study architecture and research workflow.

The overall architecture of the study and the logical progression of the research phases are summarized in the conceptual framework presented below (Figure 1).

## 2. Materials and Methods

### 2.1. Dataset Preparation

The initial phase of the study focused on establishing a robust dataset of chemicals with confirmed aneugenic activity through a bibliographic search. The search was conducted to identify chemicals known to act experimentally through an aneugenic mechanism. Papers published in the last 10 years containing at least one of the following keywords, “aneugenic” or “aneuploidy”, and “genotoxicity”, were retrieved from PubMed [14] and Scopus [15]. Regulatory documents, such as EFSA guidance documents, were also included in the search. The analysis focused on documents that included lists of aneugenic substances. This search yielded an initial list of 80 substances, each identified by its chemical name and CAS number. For entries missing the CAS number, the identifier was retrieved via PubChem.

Simplified Molecular Input Line Entry System (SMILES) strings were obtained by querying PubChem [16] using the CAS numbers and/or chemical names. The dataset underwent a rigorous curation process, which involved the exclusion of duplicates, mixtures, and substances without a well-defined structure (e.g., asbestos), resulting in a final selection of 65 unique chemicals for subsequent analysis. For entries containing salts, the structures were neutralized, and counterions were removed. Tautomeric forms and stereoisomers were addressed through expert judgment on a case-by-case basis, as the prediction tools used do not generally account for chirality.

For each substance, both chemical and toxicological information were collected (Table S1). In addition to chemical identifiers, such as Canonical SMILES, chemical name, and CAS number, Table S1 reports the results of an in-depth literature search conducted to retrieve additional specific toxicological information.

The resulting dataset of aneugens constitutes a de facto external test set for the employed profilers, as they were developed using training data from heterogeneous endpoints.

To evaluate profilers and alerts predictivity against non-aneugenic substances, a list of 38 clastogens and 47 non-genotoxic chemicals was extracted from the literature [17]. The SMILES assignment and the data curation procedure were consistent with those applied to the aneugenic dataset.

### 2.2. In Silico Profiling

The OECD QSAR Toolbox (hereinafter QSAR Toolbox) was selected as the primary platform for this study due to its high regulatory relevance and structural transparency, which are essential for supporting NAMs development and acceptance. This tool allows for the parallel application of multiple profilers across diverse datasets, providing a comprehensive screening environment. Furthermore, we leveraged its rule-based SAs to ensure that our analysis was built upon a solid mechanistic foundation, while the possibility of implementing customized features facilitated the identification and refinement of specific structure-activity relationships.

The datasets were uploaded (via their SMILES codes) into the QSAR Toolbox v. 4.8 [18]. This software is a standalone free software application developed by the Laboratory of Mathematical Chemistry (LMC) in Burgas, Bulgaria, under the coordination of the Organization for Economic Cooperation and Development (OECD) and the European Chemicals Agency (ECHA). It was designed to facilitate the practical application of (Q)SAR approaches within regulatory frameworks.

Ten mechanistic and endpoint-specific profilers, potentially relevant for genotoxicity prediction, were applied to the datasets:

- Four general mechanistic profilers: “Protein binding by OASIS”; “Protein binding by OECD”, “DNA binding by OASIS”, and “DNA binding by OECD”.
- Six endpoint-specific profilers: “DNA alerts for AMES, CA, and MNT by OASIS”; “In vitro mutagenicity (Ames test) alerts by ISS”; “In vivo mutagenicity (Micronucleus) alerts by ISS” (“In vivo MN ISS”); “Carcinogenicity (genotox and nongenotox) alerts by ISS” (“Carcinogenicity ISS”); “Protein binding alerts for Chromosomal aberration by OASIS”; and “Oncologic Primary Classification”.

Results were exported in Excel format for detailed analysis, filtering, and calculations. Among the 65 aneugenic chemicals in the initial dataset, 40 substances were identified as acting via a mechanism involving interaction with tubulin. This mechanism was the most frequently observed among the compounds in the initial dataset, as well as being the most widely studied in relation to aneugenicity.

### 2.3. Genotoxicity Databases

In the absence of specific aneugenicity databases, micronucleus assay data repositories available in the QSAR Toolbox, namely in vivo micronucleus mutagenicity results (ISSMIC) and OASIS micronucleus (OASIS MN), were employed for internal benchmarking to establish a baseline performance on the profilers. While their records do not discriminate by mode of action, they serve as a comprehensive reference set that includes both clastogens and aneugens.

ISSMIC contains 566 chemicals with critically revised in vivo MN test data, developed by Istituto Superiore di Sanità (ISS). Outcomes are categorized into positive, negative, equivocal, and inconclusive results. Details on data quality and on database development can be found elsewhere [19,20]. The stand-alone version included in the ISSTOX cluster was used, as downloaded from the ISS website [21].

The OASIS MN database includes 557 chemicals with positive and negative results obtained from the in vivo MN test [22]. The database was created and maintained by LMC. In this work, the OASIS MN version available in the QSAR Toolbox v4.8 was used.

## 3. Results and Discussion

### 3.1. State of the Art

In the literature, at least within the public domain, there is a notable absence of computational tools specifically designed for the prediction of (in vivo) aneugenicity [2]. However, models and datasets for general in vivo micronucleus prediction could be useful as references for the present study.

A number of computational tools are available for the prediction of general in vivo micronucleus induction in both proprietary and public domains. Of these, several proprietary tools have been recently evaluated for their predictive performance against a dataset of pesticides and their metabolites, showing overall poor performance of the micronucleus predictions [23,24]. Interestingly, the authors hypothesized that this result stems from the limitations of the available datasets. Indeed, the issue of data variability and uncertainty in in vivo MN test results has been recently addressed by Raitano et al. [25] through analysis of major genotoxicity repositories such as the database of the EU Reference Laboratory for alternatives to animal testing (ECVAM) [26], ISSMIC, and OASIS. The analysis reported in the paper reveals that for a large proportion of results for substances present simultaneously in two or three of these databases, the in vivo MN outcomes (expressed as positive/negative/inconclusive) differ. Other models for in vivo MN results prediction are documented in the literature and in the public domain [11,12,13,27,28]. Fan et al. [27] developed a dataset of in vivo MN results and built binary classification models using machine learning methods, reaching high performance (sensitivity in the range of 75–93%, specifically 79–91%). They were able to identify 10 structural fragments with higher frequency in MN-positive chemicals than in MN-negative chemicals. Yoo et al. [28] combined commercial QSAR platforms to construct in vivo MN models from an in-house database of non-proprietary study findings in mice. Van Bossuyt et al. [12] were able to develop QSAR models with high performance accuracy (75% with the maximum coverage, and higher at lower coverages) based on a newly implemented dataset using data collected from publicly available sources. Recently, Khondkaryan et al. also collected experimental data on MN test results in vitro and in vivo, and based on these datasets developed individual and ensemble QSAR models [12], reaching good performance for the predictions (i.e., around 70% for both sensitivity and specificity in the ensemble model for in vivo results). The data collections used to develop and validate these models were examined, where available. The findings from this analysis agree with what is reported by Raitano et al., pointing towards inherent difficulties in the reuse of in vivo MN experimental results for modeling purposes, possibly due to uncertainties and variability in the experiment itself and in results interpretation [25]. We compared three different datasets, taking ISSMIC as a reference for the comparison. Indeed, the ISSMIC database provides specific guidance-related criteria, particularly for the negative outcomes interpretation [20]. Table 1, which compares in vivo MN outcomes across the three partially overlapping datasets, namely ISSMIC with the datasets used in Fan et al. [27] and Yoo et al. [28], reveals significant discrepancies in how experimental findings are interpreted and integrated. In fact, the concordance of the results reported is remarkably low (Table 1). The data collections that were used to develop and validate these models were examined, where available, and compared with the ISSMIC database as a reference. The analysis reveals significant discrepancies in how experimental findings are interpreted and integrated, reflected in the remarkably low inter-database concordance observed for the same substances across different sources of in vivo MN outcomes (reported in Table 1).

The reported limitations, pointing to the crucial importance of experimental data quality, especially when developing and/or evaluating QSAR models based on these datasets, prompted the curation of a dataset of aneugens, ensuring a more robust basis for our analysis. Moreover, since available repositories of in vivo MN experimental outcomes generally do not specify whether results stem from a clastogenic or an aneugenic mechanism, chemicals with evidence of aneugenicity were collected ex novo for the purpose of the present study. From the literature screening, we were able to identify a set of 65 substances specifically characterized by documented aneugenicity (Table S1).

### 3.2. Predictivity of Genotoxicity Profilers

Among the tools available in the public literature, in the present study, we opted for the free QSAR Toolbox application due to the comprehensive information provided, flexibility, and transparency of results. Our decision was also informed by our hands-on experience with this tool and direct involvement in developing profilers and databases.

The primary application of the genotoxicity-relevant profilers within the QSAR Toolbox was carried out to evaluate the predictive capacity of existing models on a dataset of 65 aneugenic substances and, in parallel, to analyze the resulting SAs to identify characteristics relevant to aneuploidy. To assess profiler performance with respect to non-aneugens, the results were compared with a dataset comprising 47 non-genotoxic chemicals and 38 clastogenic substances [17].

It is crucial to note that currently, no publicly available SAR models are specific enough to predict the potential aneuploidizing effects of chemical substances. Table 2 summarizes the prediction results of the various profilers. Performance metrics were evaluated separately for aneugens (target), clastogens (mechanistic controls), and non-genotoxics (negative controls) to assess the discriminatory power of the SAs. The profilers are ranked in decreasing order of positive prediction frequency, expressed as true positive rate (TPR) or false positive rate (FPR).

$$True\;Positive\;Rate\left(TPR\right)=\frac{N.true\;positive\;results\times 100}{N.true\;positive\;results+N.false\;negative\;results}$$

$$\begin{array}{cc} False\;Positive\;Rate\left(FPR\right) & =\frac{N.false\;positive\;results\times 100}{N.false\;positive\;results+N.true\;negative\;results}=\\ & =1-specificity \end{array}$$

The endpoint-specific profilers available in the QSAR Toolbox, which are expected to detect genotoxic substances, are the “In vivo MN ISS” profiler, the “DNA alerts for AMES, CA, and MNT by OASIS” profiler, and the “Protein binding alerts for Chromosomal aberration by OASIS” profiler. However, while the “In vivo MN ISS” profiler was refined based on in vivo MN test experimental results, and is specific to this endpoint, the other two profilers address slightly different endpoints, as described in their respective QSAR Model Reporting Format (QMRF). Specifically, the “DNA alerts for AMES, CA, and MNT by OASIS” profiler investigates the presence of alerts within the target molecules, responsible for interaction with DNA, whereas the “Protein binding alerts for Chromosomal aberration by OASIS” profiler assesses the ability of target molecules to elicit clastogenicity.

As expected, performance varied significantly, with positive prediction frequencies ranging from 88% to 14%. Interestingly, the “In vivo MN ISS” profiler and the “DNA alerts for AMES, CA, and MNT by OASIS” profiler represent opposite ends of the performance spectrum, highlighting their complementarity in detecting distinct mechanisms of genotoxicity.

Overall, the results reported in Table 2 highlight (i) the lack of discriminatory power between true aneugens and non-aneugens. The “In vivo MN ISS” profiler correctly flagged 57 out of 65 substances, showing the best performance (TPR = 88%). This result is consistent with the fact that the profiler was refined on in vivo MN test results, which may include both clastogens and aneugens. However, the performance of the same profiler on the non-aneugens datasets points to a high number of false positives, as evidenced by the high TPR value on the clastogens dataset and high FPR value on non-genotoxics (Table 2).

To provide an additional benchmark, we compared the current performance of these (nominally) endpoint-specific profilers against the results obtained by applying the same profilers to the ISSMIC and OASIS MN databases, which contain in vivo MN test results (Table 3). Notably, the comparison shows that, when applied to a refined dataset of aneugenic chemicals, the sensitivity of the “In vivo MN ISS” profiler increases. Nonetheless, despite its high predictive success on the positive dataset, the “In vivo MN ISS” profiler exhibits a significant limitation, namely, high sensitivity coupled with low specificity (Table 3) [20]. In parallel, the sensitivity of the other profilers increases in databases containing both aneugens and clastogens (i.e., ISSMIC and MN OASIS), confirming their relevance for DNA-interacting mechanisms, such as those underlying clastogenicity.

The analysis of prediction results in the aneugens dataset revealed substances that consistently triggered SAs across nearly all profilers and those that triggered very few or none:

- Substances triggering few/no SAs: Only benzonitrile in the dataset did not present any SAs for any of the ten applied profilers. Furthermore, diethylstilbestrol only triggered a single alert (from the “Oncologic Primary Classification”, classifying it as a “Phenol Type Compound”).
- Substances triggering many SAs: Some chemicals triggered alerts in many applied profilers. This can be due to the presence of slightly similar alerts defined across profilers, but also to profiler alerting for different functional groups depending on the target (e.g., DNA or proteins). As an example, Melphalan triggered SAs from every profiler applied, primarily associated with the nitrogen mustard functional group. Laulimalide, Epothilone A, Trichlorfon, and Menadione triggered SAs in 9 out of the 10 profilers, targeting more than one alerting moiety.

At the same time, the observed presence of SAs known for DNA reactivity (e.g., aromatic amines or chemical mustards) in some aneugenic substances suggests the possibility of multiple modes of action, indicating that the discrimination between substances acting or not via DNA interaction is not always absolute. This aspect is critical for risk assessment, as it relates to the possibility of identifying a toxicity threshold, and will require further investigation in future studies.

Among the applied profilers on the 65 aneugenic substances, the most predictive are “In vivo MN ISS”, “Carcinogenicity ISS,” and “Oncologic Primary Classification”. With regard to “Carcinogenicity ISS”, the most prevalent SAs are “Imidazole and benzimidazole (non-genotoxic)” with a TPR of 18%, followed by “Halogenated benzene (non-genotoxic)” with a TPR of 8%. Within the “Oncologic Primary Classification” profiler, the most frequent alerts are the “Aromatic Amine Type Compounds” (TPR = 23%) and the “Carbamate Type Compounds” (TPR = 18%).

Table 4 shows the distribution of SAs from the “In vivo MN ISS” profiler, the most effective profiler in terms of the highest TPR, calculated for the aneugenic, clastogenic, and non-genotoxic substances. The top alerting fragment is codified under the “H-acceptor-path3-H-acceptor” alert. This alert encodes a structural motif potentially responsible for non-covalent interactions, which are typical of mechanisms involving aneugenic substances and their molecular targets (e.g., tubulin binding).

However, as this alert is defined by generic structural elements, it lacks the specificity required for optimal discrimination between true positives and false positives [20]. As a matter of fact, the “H-acceptor-path3-H-acceptor” alert displays high TPR values in both the aneugen dataset and the negative control list (Table 4), underlining its lack of specificity due to its ubiquitous nature. Descriptive analysis reveals a notable increase in TPR for SAs not strictly related to DNA reactivity (i.e., “H-acceptor-path3-H-acceptor” and “1,3-dialkoxy-benzene”) in the aneugens dataset compared with the others. On the other hand, alerts encoding known interactions with DNA (e.g., “alpha, beta-unsaturated carbonyls”, “Epoxides and aziridines”, and “Primary aromatic amine, hydroxyl amine and its derived esters”) show higher TPRs in the clastogens list and in the ISSMIC database. These results point to the suitability of certain alerts within the “In vivo MN ISS” profiler for aneugenicity, compared to those optimized for detecting DNA-interacting chemicals, such as clastogens.

### 3.3. Structural Alerts Relevant for Tubulin Interaction

To further refine the SAR analysis of aneuploidy induction, the second phase of the study focused on a specific molecular initiating event (MIE) among those related to chemical aneugenesis [29]. Specifically, we investigated the structural determinants of tubulin binding, as this MIE represents the most prevalent and extensively studied mechanism, providing a robust basis for quantitative modeling and mechanistic assessment [8]. Consequently, the initial list of aneugens was screened to select only chemicals with a confirmed mechanism of action involving tubulin interactions. Through an extensive literature review, 40 chemicals were identified from the initial list of aneugens as tubulin-binding aneugens (Table 5).

The TPR for each of the QSAR Toolbox Profilers was recalculated on this subset of tubulin-binding aneugens, as displayed in Table 6. Notably, the “In vivo MN ISS” and the “Carcinogenicity ISS” profilers remain the top-performing models, consistent with the analysis of the full aneugens dataset. Overall, the pattern of TPRs, although slightly modified compared to the whole aneugens list (Table 4), does not exhibit a significant enrichment within the tubulin-binding subset. This is further supported by the Observed/Expected ratio (O/E ratio) values, where the expected value (E) is calculated proportionally to the subset size (Table 6).

Focusing on the most predictive profilers, specifically “In vivo MN ISS”, “Carcinogenicity by ISS”, “Protein binding by OASIS”, and “Oncologic Primary Classification”, we analyzed the most frequently triggered SAs. Table 7 shows the variation in the number of SAs detected when narrowing the analysis from the initial dataset of 65 aneugens to the subset of 40 tubulin binders (Table 5). For comparison, the number of SAs triggered by clastogens and non-genotoxic chemicals is also reported. For the subset of tubulin-binding aneugens, the positive predictivity (PPV) of each alert was calculated as follows:

$$PPV=\frac{N.correctly\;identified\;tubuline\;binders}{N.all\;substances\;identified}$$

As shown in Table 7, while most alerts are triggered by only a few compounds, certain notable exceptions were identified and analyzed in detail below.

#### 3.3.1. The “H-Acceptor-path3-H-acceptor” Alert

The results obtained for this alert confirm our previous discussion regarding the significance of non-covalent interactions implemented in the H-acceptor-path3-H-acceptor structural path, coupled with low general specificity and lack of discriminatory power towards aneugenicity.

#### 3.3.2. The “1,3-dialkoxy-benzene” Alert

According to Table 7, the number of substances triggering the “1,3-dialkoxy-benzene” alert within the “In vivo MN ISS” profiler remains nearly constant across both the aneugenic substances dataset and tubulin-binding subset. This consistency suggests that this alert could be specific to this type of mechanism, also confirmed by the high PPV = 88%. Indeed, the SA was identified in several aneugenic substances known to bind to tubulin, notably demonstrated by colchicine, which serves as a prototypical example of this interaction (Table 8).

**Figure 2:**
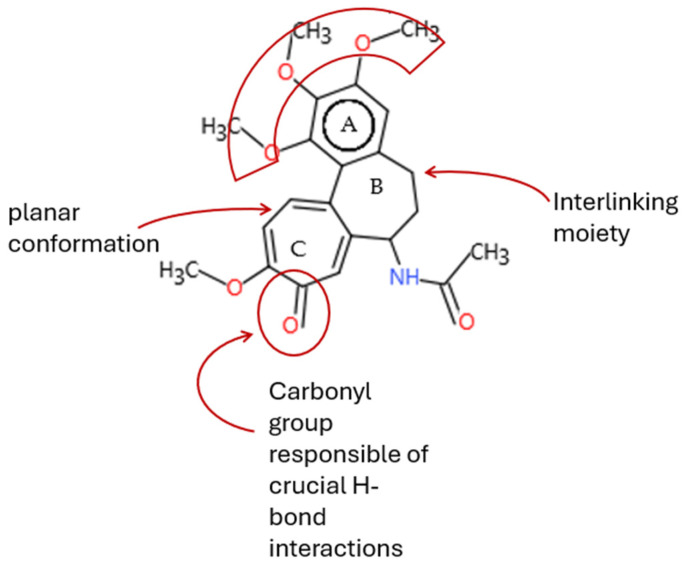
Structure of colchicine (CAS: 64-86-8) with Key Structural Portions (adapted from [8]).

An in-depth analysis of substances containing the “1,3-dialkoxy-benzene” alert was conducted using colchicine’s three structural portions (Figure 2) as a template to examine which elements were structurally compatible with tubulin binding [8].

In colchicine, the presence of the trimethoxy phenyl (Ring A) group, which gives rise to the “1,3-dialkoxy-benzene” alert, is crucial for its stable interaction with tubulin [8]. Literature studies have reiterated that the involvement of the methoxy groups is essential for ensuring the stability of the colchicine-tubulin binding [79]. While some structures included in Table 8 (e.g., Podophyllotoxin, ID: 29) maintain the trimethoxy motif (A ring), others contain 1,2 or 1,3 dimethoxy motifs, and could include other substituents (e.g., Griseofulvin, ID: 21) or cyclic alkoxy structures (e.g., Rotenone, ID: 30).

Ring B, which is a structural portion acting as a link between Rings A and C, was found to be the most variable among the structures detected by the SA. Its size and complexity vary significantly, ranging from almost absent (as in Podophyllotoxin, ID: 29) to consisting of a series of condensed rings (as in Rotenone, ID: 30).

Ring C contains a carbonyl function, known to act as a hydrogen bond acceptor, which is conserved in the corresponding structural portions of the other substances. These structural portions generally maintain a planar conformation, which appears to be critical for tubulin binding [80].

The structural features identified are important characteristics that can be implemented for the refinement of the SA. The original definition codifies generic “alkoxy” groups to describe the substituents at positions 1 and 3 of the aromatic ring. Given the precise mechanistic role of the methoxy groups in tubulin-binding stability, the original definition may be too broad. The alert could be refined by codifying at least two methoxy groups in Ring A, including the possibility of having the feature in a cycle. Moreover, to further refine the alert, a planarity requirement for the main portion of the molecule and the presence of a carbonyl group in ring C, or another H-bond acceptor, could be implemented.

#### 3.3.3. Identification of Benzimidazole-Carbamate Features as a New Potential Alert

“Imidazole and benzimidazole” alert frequently appears among the SAs triggered from the “Carcinogenicity ISS” profiler. Moreover, this alert is present in 12 substances known to bind to tubulin and induce aneugenic effects (Table 9).

The literature has highlighted the high prevalence of benzimidazole fragments in substances with positive MN test results, including herbicides belonging to the benzimidazole derivative class [27].

Of the 12 benzimidazole-containing substances reported in Table 9, 10 substances (e.g., Carbendazim, Benomyl) also trigger the “Carbamate” alert (identified by the “Oncologic Primary Classification” and the “Protein binding” alerts identified by OASIS profilers). On the other hand, there are two substances, among the tubulin-binders (i.e., Febantel and Thiophanate-methyl), triggering only the “Carbamate” alert, and not the “Imidazole and benzimidazole” one.

The literature reports suggest a possible synergistic importance of carbamate and benzimidazole substructures in the same molecule, with the latter providing reactivity and the former providing greater stability to the tubulin-ligand complex [81]. However, the present analysis highlighted that thiabendazole and triclabendazole lack the carbamate portion, instead featuring a thiazolyl moiety and a Cl-substituted benzene ring linked to the benzimidazole, respectively (IDs 32 and 35, Table 9). Since both substances are experimentally confirmed to interact with tubulin, the presence of the carbamate portion appears not to be essential. In parallel, two molecules in the list lack the benzimidazole moiety and have more than one carbamate subgroup (i.e., Thiophanate-methyl, ID: 34, and Febantel, ID: 17).

Further analysis of the substances reported in Table 9 highlights various substituents on the benzimidazole core that do not have a negative effect on reactivity. Examples of these substituents are: thioether groups present in albendazole (ID: 4) and its oxide (ID: 5); an amide moiety in Benomyl (ID: 6); a formylphenyl fragment in mebendazole (ID: 24); a thiophenoyl group in nocodazole (ID: 26).

In conclusion, a new potential SA for aneugenesis, in particular for tubulin-binding substances, containing a benzimidazole and/or a carbamate core was identified. A similar alert would have a PPV of 100% in the present subset of tubulin binders (correctly identifying 14/14 molecules). Moreover, structural features identified in the present list of substances could be codified in the SA to refine its boundaries and improve its predictivity. In this respect, the future collection of non-aneugenic substances will be crucial in the process of definition and evaluation of this new alert.

#### 3.3.4. Other Specific Structural Features

Another alert with a high PPV is the ketone functional group triggered by the “Protein binding by OASIS” profiler (PPV = 88%). While the presence of this group could be considered a warning of potential reactivity, specifically linking it to a tubulin-binding mechanism remains challenging. In the present dataset, this feature is frequently concomitant with other reactive features (e.g., the 1, 3-dialkoxy-benzene or the epoxy group). Moreover, while there are other features showing a high PPV on the tubulin-binding dataset (e.g., “alpha, beta-unsaturated carbonyls” and Epoxides), the overall number of substances triggered is insufficient to yield generalizable results. On the other hand, the presence of alerts known to involve DNA-reactive mechanisms across the entire dataset of aneugens (e.g., aromatic amines, epoxides…) suggests the potential for multiple modes of action in certain substances, in which discrimination between genotoxicity endpoints (mutation, clastogenicity, aneuploidy) is not always absolute.

#### 3.3.5. Implementation of the Structural Features into New SAs

The structural features identified in this study (i.e., based on the 1,3-dimethoxy group and benzimidazole–carbamate features) were converted into SAs by leveraging the QSAR Toolbox’s functionality to create and implement custom profilers (the corresponding encoding files are provided in the Supplementary Materials). The implemented SAs were validated against the datasets of negative controls (i.e., clastogens and non-genotoxicants), showing promising results, as reported in Table 10.

## 4. Conclusions

The present study was prompted by a significant scientific and regulatory gap: currently, no publicly available structure-activity models exist that are specifically designed to predict the aneuploidizing potential of chemical substances. This gap is accompanied by, or even driven by, a lack of curated datasets on this endpoint. In vivo MN result collections exist, but they generally lack the granularity to distinguish between clastogenic and aneugenic mechanisms. Moreover, high data variability across major databases of in vivo MN data (e.g., ISSMIC, OASIS database, ECVAM) has been recently highlighted [25].

To address these gaps and improve QSAR modeling, this study curated a new, dedicated dataset of 65 substances with specifically documented aneugenic evidence. The “In vivo MN ISS” profiler demonstrated the best performance (TPR = 88%). This is expected, as the MN test, upon which the profiler is based, is capable of detecting both clastogenic and aneugenic effects. However, the performance of the same profiler on the non-aneugens datasets points to a high number of false positive results.

A subset of 40 chemicals known to act by tubulin interaction, the most frequent and well-studied mechanism of aneugenic action, was derived from the initial dataset through an extensive literature search. Prediction results for these chemicals were further investigated in order to determine whether focusing on a single target protein altered the alert pattern.

While at the level of the overall profiler predictive capacity, the picture has only slightly changed, an in-depth analysis of the results pattern allowed the identification of promising alerts encoding key structural characteristics known to be relevant for tubulin-binding mechanisms.

The “1,3-dialkoxy-benzene” alert was found in crucial mitotic spindle poisons like colchicine, where the trimethoxy phenyl moiety is fundamental for tubulin interaction and binding stability. The present analysis proposed a more refined definition of this alert to more accurately reflect the structural requirements for tubulin binding. Moreover, the benzimidazole and carbamate structural features were identified as key characteristics (in combination or alone) for a new SAs specifically detecting tubulin-binding aneugens.

Notably, the presence of SAs typically associated with DNA reactivity (e.g., aromatic amines, epoxides) in various aneugenic substances (such as Melphalan, Laulimalide, Epothilone A) suggests potential multiple modes of action. This indicates that the boundaries between genotoxicity endpoints (gene mutation, clastogenicity, and aneuploidy) are not always absolute, requiring further investigation for robust hazard and risk assessment.

Moreover, this study emphasizes the crucial importance of well-curated data in unraveling the structural determinants of toxicity. Incorporating key structural characteristics from high-quality data into profilers implemented in public domain software provides regulators with useful tools for early hazard assessment. For instance, these tools can directly inform the prediction of the MIE, specifically chemical binding to tubulin, as defined in “The adverse outcome pathway (AOP) for Chemical Binding to Tubulin in Oocytes Leading to Aneuploid Offspring” [8].

Our findings underscore the significant potential of NAMs, specifically in silico SAR-based models, pointing to the necessity of expanding current datasets with additional chemicals with known aneugenic mechanisms, as well as those for which aneugenic potential can be definitively excluded. Since these types of substances are extensively studied as key pharmacological tools in drug discovery, the study demonstrates how such mechanistic insights can be exploited to build a comprehensive list of SAs for predictive toxicology.

Early in silico prediction of aneugenicity would represent a major advancement in reducing animal testing, aligning with the 3Rs principles. Moreover, it would support the definition of regulatory thresholds, which are essential for the risk assessment of aneugenic substances.

## Figures and Tables

**Table 1:** Comparison of outcomes across three partially overlapping datasets. The number of chemicals in the ISSMIC database is 566.

N. of Chemicals	Yoo et al. [28]	Fan et al. [27]
Total number of chemicals	1131	576
Number of chemicals overlapping with the ISSMIC database	438	100
Outcomes concordance	49%	54%

**Table 2:** Number of substances with SA and corresponding predictivity (expressed as TPR or FPR) for selected QSAR Toolbox Profilers. Data are grouped into three mechanistic classes: 65 aneugens, 38 clastogens, and 47 non-genotoxic substances.

	Aneugens		Clastogens		Non-Genotoxics	
QSAR Toolbox Profilers	N Subs with SA	TPR (%)	N Subs with SA	TPR (%)	N Subs with SA	FPR (%)
In vivo MN ISS	57	88%	35	92%	35	74%
Carcinogenicity ISS	43	66%	29	76%	20	43%
Oncologic Primary Classification	43	66%	26	68%	20	43%
DNA binding by OECD	30	46%	27	71%	21	45%
Protein binding by OASIS	30	46%	20	53%	19	40%
Protein binding by OECD	27	42%	19	50%	17	36%
In vitro mutagenicity (Ames test) alerts by ISS	19	29%	27	71%	12	26%
Protein binding alerts for Chromosomal aberration by OASIS	18	28%	18	47%	6	13%
DNA binding by OASIS	13	20%	25	66%	6	13%
DNA alerts for AMES, CA, and MNT by OASIS	9	14%	22	58%	2	4%

**Table 3:** Sensitivity (SE), Specificity (SP) of three QSAR Toobox profilers (“DNA alerts for AMES, CA, and MNT by OASIS”, “Protein binding alerts for Chromosomal aberration by OASIS” and “In vivo MN ISS” profilers) versus ISSMIC and OASIS databases.

	ISSMIC Database		MN OASIS Database	
Profilers	SE	SP	SE	SP
DNA alerts for AMES, CA, and MNT by OASIS	28%	82%	40%	70%
Protein binding alerts for Chromosomal aberration by OASIS	26%	75%	38%	73%
In vivo MN ISS	70%	30%	82%	29%

**Table 4:** Distribution of SAs from “In vivo MN ISS” profiler, including TPR and FPR percentages calculated for the aneugenic, clastogenic, and non-genotoxic substances.

SAs In vivo MN ISS Profiler	Aneugens N SAs (TPR%)	Clastogens N SAs (TPR%)	Non-Genotoxics N SAs (FPR%)
H-acceptor-path3-H-acceptor	55 (85%)	29 (76%)	29 (62%)
1,3-dialkoxy-benzene	8 (12%)	1 (3%)	1 (2%)
alpha, beta-unsaturated carbonyls	4 (6%)	5 (13%)	4 (9%)
Epoxides and aziridines	3 (5%)	4 (11%)	0
Oxolane	3 (5%)	3 (8%)	3 (6%)
1-phenoxy-benzene	2 (3%)	0	0
Nitro-aromatic	2 (3%)	2 (5%)	0
Primary aromatic amine, hydroxyl amine and its derived esters	2 (3%)	0	2 (4%)
Quinones	2 (3%)	6 (16%)	0
S or N mustard	1 (2%)	2 (5%)	0
Alkyl (C < 5) or benzyl ester of sulphonic or phosphonic acid	1 (2%)	2 (5%)	0
Hydrazine	1 (2%)	1 (3%)	1 (2%)
Azide and triazene groups	0	3 (8%)	0
Alkyl carbamate and thiocarbamate	0	2 (5%)	1 (2%)
Alkyl and aryl N-nitroso groups	0	2 (5%)	0
Propiolactones or propiosultones	0	1 (3%)	0
Aromatic ring N-oxide	0	1 (3%)	0
Aliphatic N-nitro group	0	1 (3%)	0

**Table 5:** List of 40 aneugenic substances, with confirmed evidence in the literature of interaction with tubulin.

ID	Chemical Name	CAS Number	References
1	2-methoxyestradiol	362-07-2	[30,31,32]
2	Acetaldehyde	75-07-0	[33]
3	Acrylonitrile	107-13-1	[34]
4	Albendazole	54965-21-8	[35]
5	Albendazole oxide	54029-12-8	[35]
6	Benomyl	17804-35-2	[36]
7	Benzonitrile	100-47-0	[37,38]
8	Carbendazim	10605-21-7	[35]
9	Chloral Hydrate	302-17-0	[39]
10	Colcemid	108964-31-4	[40]
11	Colchicine	64-86-8	[39]
12	Crizotinib	877399-52-5	[41,42]
13	Diazepam	439-14-5	[39,43,44]
14	Econazole	27220-47-9	[45]
15	Epothilone A	152044-53-6	[46,47]
16	Etoposide	33419-42-0	[48,49]
17	Febantel	58306-30-2	[50]
18	Fenbendazole	43210-67-9	[51]
19	Fenvalerate	51630-58-1	[52,53]
20	Flubendazole	31430-15-6	[35]
21	Griseofulvin	126-07-8	[54,55,56]
22	Ixabepilone	219989-84-1	[57]
23	Laulimalide	115268-43-4	[58]
24	Mebendazole	31431-39-7	[35]
25	Menadione	58-27-5	[59]
26	Nocodazole	31430-18-9	[55,60,61]
27	Oxfendazole	53716-50-0	[51,62]
28	Oxibendazole	20559-55-1	[35]
29	Podophyllotoxin	518-28-5	[63,64]
30	Rotenone	83-79-4	[65,66]
31	Taxol	33069-62-4	[67]
32	Thiabendazole	148-79-8	[35]
33	Thiocolchicoside	602-41-5	[68,69]
34	Thiophanate-methyl	23564-05-8	[70]
35	Triclabendazole	68786-66-3	[71,72]
36	Vinblastine	865-21-4	[73]
37	Vincristine	57-22-7	[74]
38	Vindesine	53643-48-4	[75,76]
39	Vinflunine	162652-95-1	[77]
40	Vinorelbine	71486-22-1	[78]

**Table 6:** Predictivity (reported as TPR) of QSAR Toolbox Profilers (v 4.8) on 40 Aneugenic Substances with evidence of tubulin binding. O/E ratio: Observed/Expected ratio.

QSAR Toolbox Profilers	N Substances with SA	TPR (%)	O/E Ratio
In vivo MN ISS	37	93%	1.06
Carcinogenicity ISS	29	73%	1.12
Protein binding by OASIS	25	63%	1.39
Oncologic Primary Classification	23	58%	0.88
DNA binding by OECD	17	43%	0.94
Protein binding by OECD	16	40%	0.94
Protein binding alerts for Chromosomal aberration by OASIS	14	35%	1.27
In vitro mutagenicity (Ames test) alerts by ISS	11	28%	0.92
DNA binding by OASIS	8	20%	1.00
DNA alerts for AMES, CA, and MNT by OASIS	5	13%	0.83

**Table 7:** Variation in the number and percentage of SAs for four QSAR Toolbox Profilers (i.e., “In vivo MN ISS”, “Carcinogenicity ISS”, “Oncologic Primary Classification”, and “Protein binding by OASIS” profilers), when moving from 65 aneugenic substances to 40 tubulin-binding substances; comparison with clastogens and non-genotoxic chemicals. Only occurrences of more than two SAs for the 65 substances are reported. PPV: positive predictivity.

QSAR Toolbox Profilers	Structural Alerts	N SAs on 65 Aneugenic Substances	N SAs on 40 Tubulin-Binding Substances (PPV)
In vivo MN ISS	H-acceptor-path3-H-acceptor	55	36 (66%)
	1,3-dialkoxy-benzene	8	7 (88%)
	alpha, beta-unsaturated carbonyls	4	4 (100%)
	Epoxides and aziridines	3	3 (100%)
	Oxolane	3	2 (67%)
Carcinogenicity ISS	Imidazole, benzimidazole (Nongenotox)	12	12 (100%)
	Halogenated benzene (Nongenotox)	5	2 (40%)
	alpha, beta-unsaturated carbonyls (Genotox)	4	4 (100%)
	Epoxides and aziridines (Genotox)	3	3 (100%)
	1,3-Benzodioxoles (Nongenotox)	3	2 (67%)
Oncologic Primary Classification	Aromatic Amine Type Compounds	15	3 (20%)
	Carbamate Type Compounds	12	12 (100%)
	Phenol Type Compounds	8	2 (25%)
	Epoxide Reactive Functional Groups	3	3 (100%)
Protein binding by OASIS	Carbamates and N-substituted carbamates	12	12 (100%)
	Ketones	8	7 (88%)
	Epoxides, Aziridines, and Sulfuranes	3	3 (100%)
	Activated alkyl esters and thioesters	3	3 (100%)
	Conjugated systems with electron-withdrawing groups	3	3 (100%)

**Table 8:** Substances triggering the “1,3-dialkoxy-benzene” alert, included in the “In vivo MN ISS” Profiler.

ID	Chemical Name (CAS)	Structure Triggering 1,3-Dialkoxy-benzene Alert
10	Colcemid (108964-31-4)	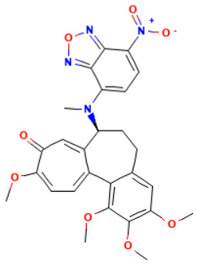
11	Colchicine (64-86-8)	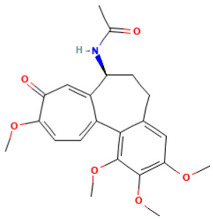
16	Etoposide (33419-42-0)	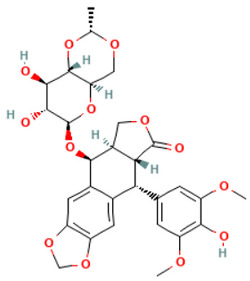
21	Griseofulvin (126-07-8)	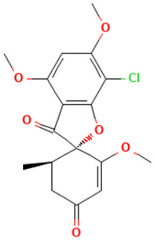
29	Podophyllotoxin (518-28-5)	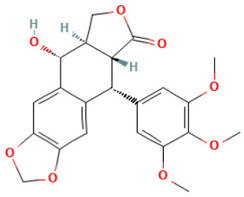
30	Rotenone (83-79-4)	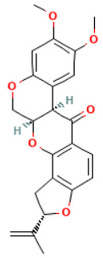
33	Tiocolchicoside (602-41-5)	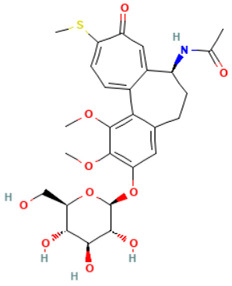

**Table 9:** Substances triggering the “Imidazole and benzimidazole” alert, included in the “Carcinogenicity by ISS” and/or “Carbamate” alert, included in the “Oncologic Primary Classification” (from QSAR Toolbox 4.8).

ID	Chemical Name (CAS)	Chemical Structure	Imidazole and Benzimidazole SA (Carc ISS)	Carbamate SA (Oncologic Primary Classification)
4	Albendazole (54965-21-8)	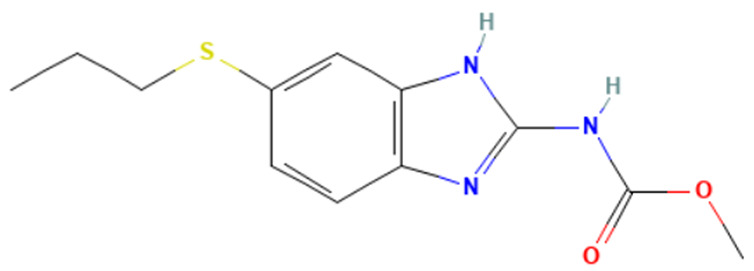	yes	yes
5	Albendazole oxide (54029-12-8)	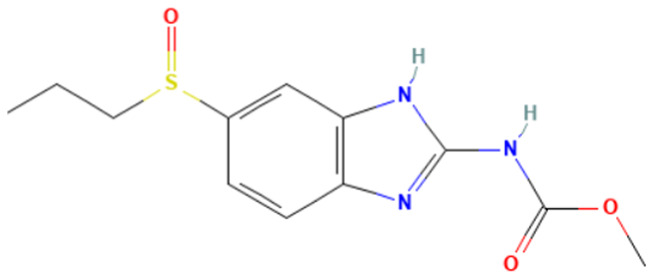	yes	yes
6	Benomyl (17804-35-2)	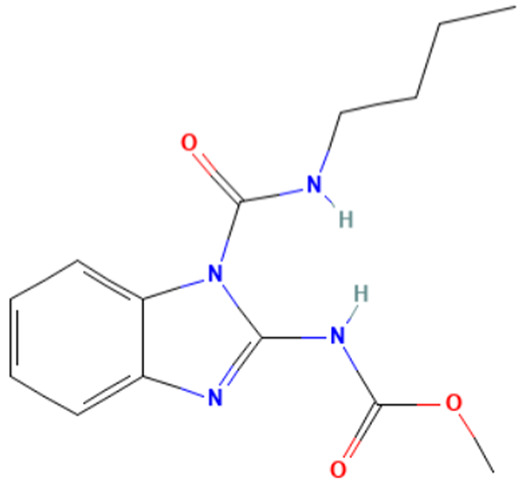	yes	yes
8	Carbendazim (10605-21-7)	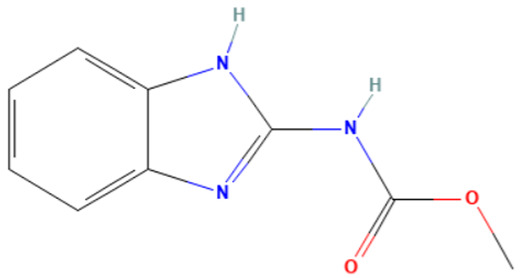	yes	yes
17	Febantel (58306-30-2)	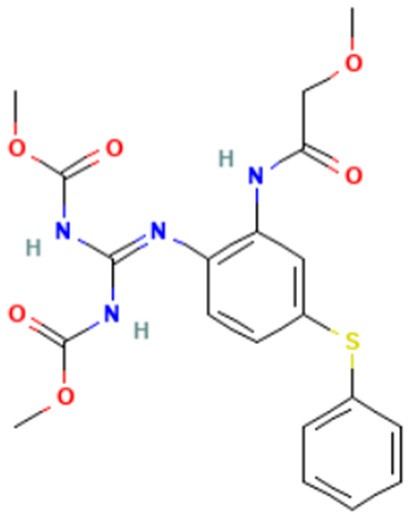	no	yes
19	Fenbendazole (43210-67-9)	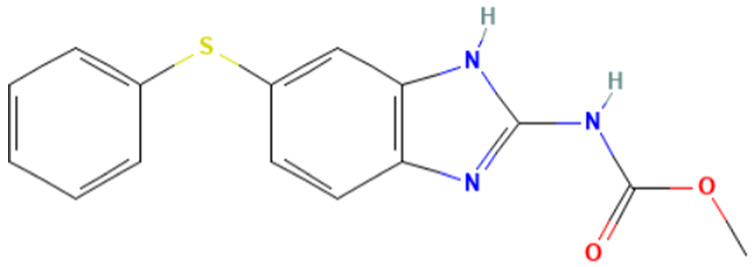	yes	yes
20	Flubendazole (31430-15-6)	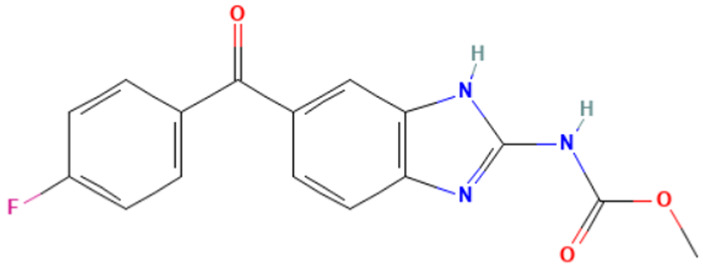	yes	yes
24	Mebendazole (31431-39-7)	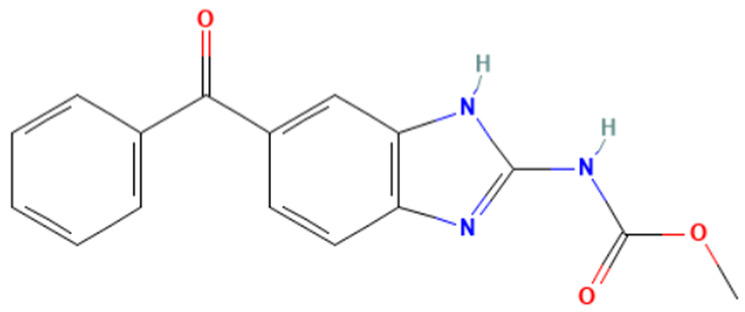	yes	yes
26	Nocodazole (31430-18-9)	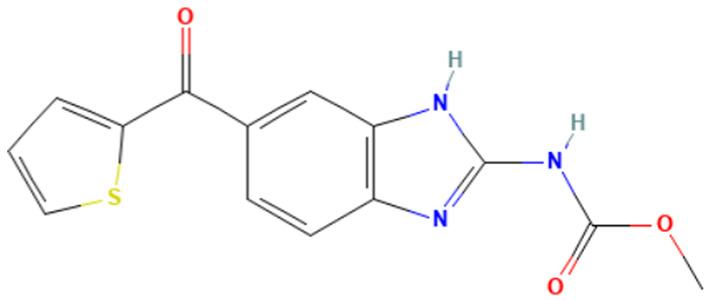	yes	yes
27	Oxfendazole (53716-50-0)	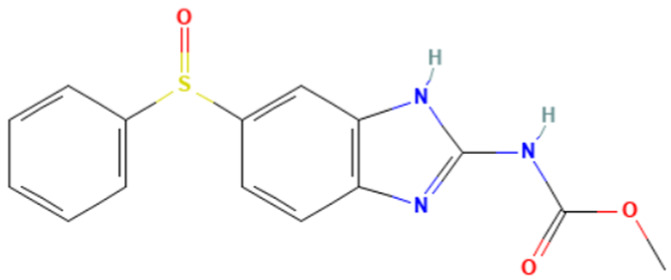	yes	yes
28	Oxibendazole (20559-55-1)	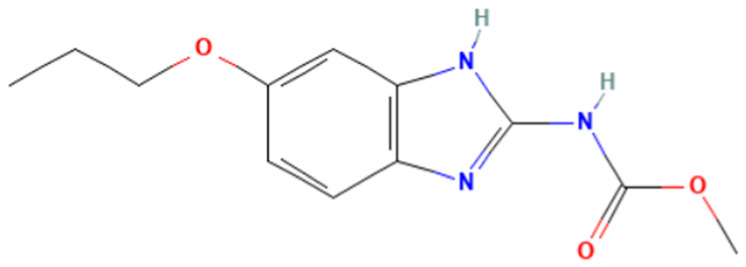	yes	yes
32	Thiabendazole (148-79-8)	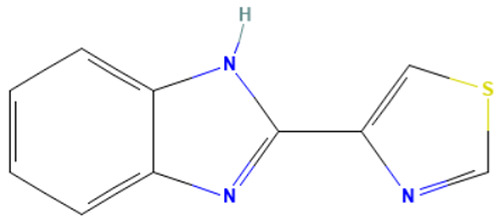	yes	no
34	Thiophanate-methyl (23564-05-8)	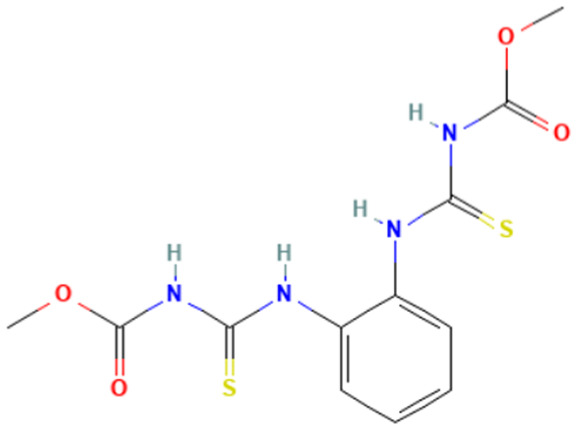	no	yes
35	Triclabendazole (68786-66-3)	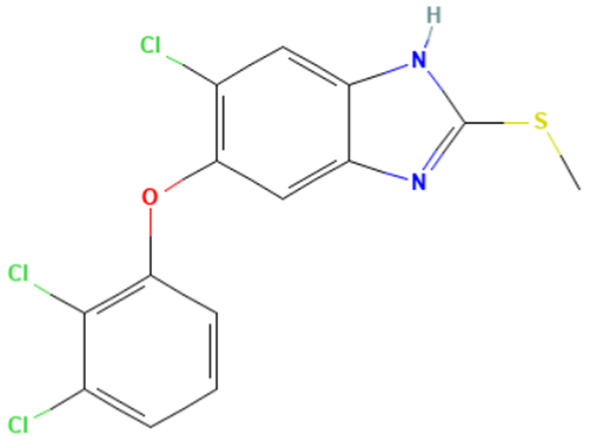	yes	no

**Table 10:** Distribution of substances across different mechanistic classes in relation to the new SAs identified in this study. SA1: 1,3 dimethoxy group; SA2: Benzimidazole-Carbamate features.

	N SAs on 40 Tubulin-Binding Substances (TPR)	N SAs on 38 Clastogens (TPR)	N SAs on 47 Non-Genotoxics (FPR)
SA1	14 (100%)	0	1 (2%)
SA2	7 (100%)	0	0

## Data Availability

The data used in this study are publicly available in the sources cited in the Section 2. The processed dataset used is available in the Supplementary Material (Table S1).

## Supplementary Materials

The following supporting information can be downloaded at: https://www.mdpi.com/article/10.3390/toxics14050363/s1, Table S1: list of the 65 substances with evidence of aneugenic activity, compiled through an in-depth literature review. The list includes the following information for each chemical: chemical name, CAS number, canonical SMILES, evidence of tubulin-binding, and literature references. 2 SAs encoding files (SA1 Carbamate_Benzimidazole; SA2 Colchicine_like alert) [30,31,32,33,34,35,36,37,38,39,40,41,42,45,46,48,49,50,51,52,53,54,55,56,57,58,59,60,61,62,63,64,65,66,67,68,69,70,71,72,73,74,75,77,78,82,83,84,85,86,87,88,89,90,91,92,93,94,95,96,97,98,99,100,101,102,103,104,105,106,107,108,109,110,111,112,113,114].

## Abbreviations

The following abbreviations are used in this manuscript:

AOP	Adverse Outcome Pathway
3R principles	Reduction, Refinement, Replacement principles
“Carcinogenicity ISS” profiler	“Carcinogenicity (genotox and non-genotox) alerts by ISS” profiler
CREST	Kinetochore staining
ECVAM	EU Reference Laboratory for alternatives to animal testing
EFSA	European Food Safety Authority
FISH	Fluorescence in situ hybridization
FPR	False Positive Rate
ISS	Istituto Superiore di Sanità
ISSMIC database	In vivo MN test database developed by ISS
LD50	Lethal dose, 50%
LMC	Laboratory of Mathematical Chemistry in Burgas, Bulgaria
“In vivo MN ISS” profiler	“In vivo mutagenicity (micronucleus) alerts by ISS” profiler
NAMs	New Approach Methodologies
MIE	Molecular Initiating Event
MN Test	Micronucleus Test
OASIS MN database	In vivo MN test database developed by LMC
PPV	Positive Predictivity
QMRF	QSAR Model Reporting Format
QSAR	Quantitative Structure-Activity Relationship
SAR	Structure-Activity Relationship
SAs	Structural Alerts
SE	Sensitivity
SMILES	Simplified molecular input-line entry system
SP	Specificity
TPR	True Positive Rate
