# Supplementary material for: Structural Alerts for Aneuploidy Prediction: Are We There Yet?
Source: Toxics. 2026 Apr 24;14(5):363. doi: 10.3390/toxics14050363 (PMC13211754; doi:10.3390/toxics14050363)
Supplement: Supplementary file 1 [file toxics-14-00363-s001.zip › toxics-4223332-supplementary_Table_S1.pdf]

Table S1. List of 65 substances with evidence of aneugenic activity, compiled through an in-depth literature review. The list includes the following information for each chemical: Chemical name, CAS number, Canonical SMILES, Evidence of tubulin binding and literature references.

Chemical Name	CAS Number	SMILES	Tubulin binding	References
17Beta-estradiol	50-28-2	CC12CCC3C(CCc4cc(O)ccc34)C1CCC2O	no	[1]
2-methoxyestradiol	362-07-2	COc1cc2C3CCC4(C)C(O)CCC4C3CCc2cc1O	yes	[2–4]
3-(1H-Indazol-5-yl)-N-propylimidazo(1,2-b)pyridazin-6-amine (CHR-6494)	1333377-65-3	CCCNc1ccc2ncc(-c3ccc4[nH]ncc4c3)n2n1	no	[5]
4-(4-(N-benzoylamino)anilino)-6-methoxy-7-(3-(1-morpholino)propoxy)quinazoline (ZM447439)	331771-20-1	COc1cc2c(Nc3ccc(NC(=O)c4ccccc4)cc3)ncnc2cc1OCCCN1CCOCC1	no	[6]
Acetaldehyde	75-07-0	CC=O	yes	[7]
Acetamide, N-[2-[6-[[4-(cyclobutylamino)-5-(trifluoromethyl)-2-pyrimidinyl]amino]-1,2,3,4-tetrahydronaphthalen-1,4-imin-9-yl]-2-oxoethyl]- (PF-03814735)	942487-16-3	CC(=O)NCC(=O)N1C2CCC1c1cc(Nc3ncc(c(NC4CCC4)n3)C(F)(F)F)ccc21	no	[8]
Acrylonitrile	107-13-1	C=CC#N	yes	[9]
Albendazole	54965-21-8	CCCSc1ccc2nc(NC(=O)OC)[nH]c2c1	yes	[10]
Albendazole oxide	54029-12-8	CCC[S+]([O-])c1ccc2nc(NC(=O)OC)[nH]c2c1	yes	[10]
Alisertib (MLN 8237)	1028486-01-2	COc1cccc(F)c1C1=NCc2cnc(Nc3ccc(C(O)=O)c(OC)c3)nc2-c2ccc(Cl)cc12	no	[11,12]
Amsacrine	51264-14-3	COc1cc(NS(C)(=O)=O)ccc1Nc1c2ccccc2nc2ccccc12	no	[13]
Barasertib	722544-51-6	CCN(CCO)CCCOc1ccc2c(Nc3cc(CC(=O)Nc4cccc(F)c4)[nH]n3)ncnc2c1	no	[14]
Benomyl	17804-35-2	CCCCNC(=O)n1c(NC(=O)OC)nc2ccccc21	yes	[15]
Benzonitrile	100-47-0	N#Cc1ccccc1	yes	[16,17]
Bisphenol A	80-05-7	CC(C)(c1ccc(O)cc1)c1ccc(O)cc1	no	[1]
Carbendazim	10605-21-7	COC(=O)Nc1nc2ccccc2[nH]1	yes	[10]
Carbonyl cyanide (m-	555-60-2	Clc1cccc(NN=C(C#N)C#N)c1	unknown	[18]

chlorophenyl)hydrazone (CCCP)				
Chloral Hydrate	302-17-0	OC(O)C(Cl)(Cl)Cl	yes	[19–21]
Colcemid	108964-31-4	COC1=CC=C2C(=CC1=O)C(CCc1cc(OC)c(OC)c(OC)c12)N(C)c1ccc(c2nonc12)[N+]([O-])=O	yes	[22]
Colchicine	64-86-8	COC1=CC=C2C(=CC1=O)C(CCc1cc(OC)c(OC)c(OC)c12)NC(C)=O	yes	[19]
Crizotinib	877399-52-5	CC(Oc1cc(cnc1N)-c1cnn(c1)C1CCNCC1)c1c(Cl)ccc(F)c1Cl	yes	[23,24]
Danusertib (PHA-739358)	827318-97-8	COC(C(=O)N1Cc2[nH]nc(NC(=O)c3ccc(cc3)N3CCN(C)CC3)c2C1)c1ccccc1	no	[25]
Di(2-ethylhexyl) phthalate (DEHP)	117-81-7	CCCCC(CC)COC(=O)c1ccccc1C(=O)OCC(CC)CCCC	unknown	[26]
Diazepam	439-14-5	CN1C(=O)CN=C(c2ccccc2)c2cc(Cl)ccc12	yes	[19]
Diethylstilbestrol	56-53-1	CCC(=C(CC)c1ccc(O)cc1)c1ccc(O)cc1	no	[1]
Econazole	27220-47-9	Clc1ccc(COC(Cn2ccnc2)c2ccc(Cl)cc2Cl)cc1	yes	[27]
Epirubicin	56420-45-2	COc1cccc2C(=O)c3c(O)c4CC(O)(CC(OC5CC(N)C(O)C(C)O5)c4c(O)c3C(=O)c12)C(=O)CO	no	[13]
Epothilone A	152044-53-6	CC1CCCC2OC2CC(OC(=O)CC(O)C(C)(C)C(=O)C(C)C1O)C(C)=Cc1csc(C)n1	yes	[28]
Esfenvalerate	66230-04-4	CC(C)C(C(=O)OC(C#N)c1cccc(Oc2ccccc2)c1)c1ccc(Cl)cc1	yes	[29,30]
Etoposide	33419-42-0	COc1cc(cc(OC)c1O)C1C2C(COC2=O)C(OC2OC3COC(C)OC3C(O)C2O)c2cc3OCOc3cc12	yes	[31,32]
Febantel	58306-30-2	COCC(=O)Nc1cc(Sc2ccccc2)ccc1N=C(NC(=O)OC)NC(=O)OC	yes	[33]
Fenbendazole	43210-67-9	COC(=O)Nc1nc2ccc(Sc3ccccc3)cc2[nH]1	yes	[34]
Flubendazole	31430-15-6	COC(=O)Nc1nc2ccc(cc2[nH]1)C(=O)c1ccc(F)cc1	yes	[10]
Griseofulvin	126-07-8	COc1cc(OC)c2C(=O)C3(Oc2c1Cl)C(C)CC(=O)C=C3OC	yes	[35–37]
Hesperadin	422513-13-1	CCS(=O)(=O)Nc1ccc2[nH]c(O)c(C(=Nc3ccc(CN4CCCCC4)cc3)c3ccccc3)c2c1	no	[38]
Ixabepilone	219989-84-1	CC1CCCC2(C)OC2CC(NC(=O)CC(O)C(C)(C)C(=O)C(C)C1O)C(C)=Cc1csc(C)n1	yes	[39]
Laulimalide	115268-43-4	CC1CC2CC=CC(CC=CC(=O)OC(CC3OC3C(O)CC(=C)C1)C(O)C=CC1CC(C)=CCO1)O2	yes	[40,41]
Mebendazole	31431-39-7	COC(=O)Nc1nc2ccc(cc2[nH]1)C(=O)c1ccccc1	yes	[10]
Melphalan	148-82-3	NC(Cc1ccc(cc1)N(CCCl)CCCl)C(O)=O	no	[42]
Menadione	58-27-5	CC1=CC(=O)c2ccccc2C1=O	yes	[43]

Merbarone	97534-21-9	O=C(Nc1ccccc1)C1C(=O)NC(=S)NC1=O	no	[13,44,45]
Mono(2-ethylhexyl) phthalate (MEHP)	4376-20-9	CCCCC(CC)COC(=O)c1ccccc1C(O)=O	unknown	[46]
N-(4-((3-(2-Amino-4-pyrimidinyl)-2-pyridinyl)oxy)phenyl)-4-(4-methyl-2-thienyl)-1-phthalazinamine (AMG-900)	945595-80-2	Cc1csc(c1)-c1nnc(Nc2ccc(Oc3ncccc3-c3ccnc(N)n3)cc2)c2ccccc12	no	[47,48]
Nitrilotriacetic acid	139-13-9	OC(=O)CN(CC(O)=O)CC(O)=O	no	[49]
Nitrobenzene	98-95-3	[O-][N+](=O)c1ccccc1	no	[50,51]
Nocodazole	31430-18-9	COC(=O)Nc1nc2ccc(cc2[nH]1)C(=O)c1cccs1	yes	[37,52,53]
Oxfendazole	53716-50-0	COC(=O)Nc1nc2ccc(cc2[nH]1)[S+]([O-])c1ccccc1	yes	[34,54]
Oxibendazole	20559-55-1	CCCOc1ccc2nc(NC(=O)OC)[nH]c2c1	yes	[10]
Phenolphthalein	77-09-8	Oc1ccc(cc1)C1(OC(=O)c2ccccc21)c1ccc(O)cc1	no	[55,56]
Podophyllotoxin	518-28-5	COc1cc(cc(OC)c1OC)C1C2C(COC2=O)C(O)c2cc3OCOc3cc12	yes	[57–59]
Rotenone	83-79-4	COc1cc2OC[C@H]3Oc4c(ccc5O[C@H](Cc54)C(C)=C)C(=O)[C@H]3c2cc1OC	yes	[60,61]
Taxol	33069-62-4	CC(=O)O[C@H]1C(=O)[C@]2(C)[C@@H](O)C[C@H]3OC[C@@]3(OC(C)=O)[C@H]2[C@H](OC(=O)c2ccccc2)[C@]2(O)C[C@H](OC(=O)[C@H](O)[C@@H](NC(=O)c3ccccc3)c3ccccc3)C(C)=C1C2(C)C	yes	[62,63]
Teniposide	29767-20-2	COc1cc(cc(OC)c1O)C1C2C(COC2=O)C(OC2OC3COC(OC3C(O)C2O)c2cccs2)c2cc3OCOc3cc12	no	[13]
Thiabendazole	148-79-8	c1ccc2[nH]c(nc2c1)-c1cscn1	yes	[10]
Thiocolchicoside	602-41-5	COc1c(OC2OC(CO)C(O)C(O)C2O)cc2CCC(NC(C)=O)C3=CC(=O)C(SC)=CC=C3c2c1OC	yes	[64,65]
Thiophanate-methyl	23564-05-8	COC(=O)NC(=S)Nc1ccccc1NC(=S)NC(=O)OC	yes	[66]
Tozasertib (VX-680)	639089-54-6	CN1CCN(CC1)c1cc(Nc2cc(C)[nH]n2)nc(Sc2ccc(NC(=O)C3CC3)cc2)n1	no	[67,68]
Trichlorfon	52-68-6	COP(=O)(OC)C(O)C(Cl)(Cl)Cl	unknown	[69,70]
Triclabendazole	68786-66-3	CSc1nc2cc(Oc3cccc(Cl)c3Cl)c(Cl)cc2[nH]1	yes	[71,72]
Vinblastine	865-21-4	CCC1(O)CC2CN(CCc3c([nH]c4ccccc43)C(C2)(C(=O)OC)c2cc3c(cc2OC)N(C)C2C(O)(C(OC(C)=O)C4(CC)C=CCN5CCC23C54)C(=O)OC)C1	yes	[73]
Vincristine	57-22-7	CCC1(O)CC2CN(CCc3c([nH]c4ccccc43)C(C2)(C(=O)OC)c2cc3c(cc2OC)N(C=O)C2C(O)(C(OC(C)=O)C4(CC)C=CCN5CCC23C54)C(=O)OC)C1	yes	[74]

Vindesine	53643-48-4	CCC1(O)CC2CN(CCc3c([nH]c4ccccc43)C(C2)(C(=O)OC)c2cc3c(cc2OC)N(C)C2C(O)(C(O)C4(CC)C=CCN5CCC23C54)C(N)=O)C1	yes	[75]
Vinflunine	162652-95-1	CCC12C=CCN3CCC4(C(N(C)c5cc(OC)c(cc54)C4(CC5CC(CN(C5)Cc5c4[nH]c4ccccc45)C(C)(F)F)C(=O)OC)C(O)(C1OC(C)=O)C(=O)OC)C32	yes	[76]
Vinorelbine	71486-22-1	CCC1CN2CC(CC(C(=O)OC)(c3cc4c(cc3OC)N(C)C3C(O)(C(OC(C)=O)C5(CC)C=CCN6CCC34C65)C(=O)OC)c3[nH]c4ccccc4c3C2)C=1	yes	[77]
Volasertib	755038-65-4	CCC1N(C(C)C)c2nc(Nc3ccc(cc3OC)C(=O)NC3CCC(CC3)N3CCN(CC4CC4)CC3)ncc2N(C)C1=O	no	[78,79]

References

1. Kabil, A.; Silva, E.; Kortenkamp, A. Estrogens and Genomic Instability in Human Breast Cancer Cells--Involvement of Src/Raf/Erk Signaling in Micronucleus Formation by Estrogenic Chemicals. *Carcinogenesis* **2008**, *29*, 1862–1868, doi:10.1093/carcin/bgn138.

2. Attalla, H.; Mäkelä, T.P.; Adlercreutz, H.; Andersson, L.C. 2-Methoxyestradiol Arrests Cells in Mitosis without Depolymerizing Tubulin. *Biochem. Biophys. Res. Commun.* **1996**, *228*, 467–473, doi:10.1006/bbrc.1996.1683.

3. Gökmen-Polar, Y.; Escuin, D.; Walls, C.D.; Soule, S.E.; Wang, Y.; Sanders, K.L.; LaVallee, T.M.; Wang, M.; Guenther, B.D.; Giannakakou, P.; et al. β-Tubulin Mutations Are Associated with Resistance to 2-Methoxyestradiol in MDA-MB-435 Cancer Cells. *Cancer Res.* **2005**, *65*, 9406–9414, doi:10.1158/0008-5472.CAN-05-0088.

4. Sun, M.; Zhang, Y.; Qin, J.; Ba, M.; Yao, Y.; Duan, Y.; Liu, H.; Yu, D. Synthesis and Biological Evaluation of New 2-Methoxyestradiol Derivatives: Potent Inhibitors of Angiogenesis and Tubulin Polymerization. *Bioorg. Chem.* **2021**, *113*, 104988, doi:10.1016/j.bioorg.2021.104988.

5. Wang, P.; Hua, X.; Bryner, Y.H.; Liu, S.; Gitter, C.B.; Dai, J. Haspin Inhibition Delays Cell Cycle Progression through Interphase in Cancer Cells. *J. Cell. Physiol.* **2020**, *235*, 4508–4519, doi:10.1002/jcp.29328.

6. Kaestner, P.; Stolz, A.; Bastians, H. Determinants for the Efficiency of Anticancer Drugs Targeting Either Aurora-A or Aurora-B Kinases in Human Colon Carcinoma Cells. *Mol. Cancer Ther.* **2009**, *8*, 2046–2056, doi:10.1158/1535-7163.MCT-09-0323.

7. TUMA, D.J.; SMITH, S.L.; SORRELL, M.F. Acetaldehyde and Microtubules [a]. *Ann. N. Y. Acad. Sci.* **1991**, *625*, 786–792, doi:10.1111/j.1749-6632.1991.tb33920.x.

8. Jani, J.P.; Arcari, J.; Bernardo, V.; Bhattacharya, S.K.; Briere, D.; Cohen, B.D.; Coleman, K.; Christensen, J.G.; Emerson, E.O.; Jakowski, A.; et al. PF-03814735, an Orally Bioavailable Small Molecule Aurora Kinase Inhibitor for Cancer Therapy. *Mol. Cancer Ther.* **2010**, *9*, 883–894, doi:10.1158/1535-7163.MCT-09-0915.

9. Perin, N.; Hok, L.; Beč, A.; Persoons, L.; Vanstreels, E.; Daelemans, D.; Vianello, R.; Hranjec, M. N-Substituted Benzimidazole Acrylonitriles as in Vitro Tubulin Polymerization Inhibitors: Synthesis, Biological Activity and Computational Analysis. *Eur. J. Med. Chem.* **2021**, *211*, 113003, doi:10.1016/j.ejmech.2020.113003.

10. Ermler, S.; Scholze, M.; Kortenkamp, A. Seven Benzimidazole Pesticides Combined at Sub-Threshold Levels Induce Micronuclei in Vitro. *Mutagenesis* **2013**, *28*, 417–426, doi:10.1093/mutage/get019.

11. Manfredi, M.G.; Ecsedy, J.A.; Chakravarty, A.; Silverman, L.; Zhang, M.; Hoar, K.M.; Stroud, S.G.; Chen, W.; Shinde, V.; Huck, J.J.; et al. Characterization of Alisertib (MLN8237), an Investigational Small-Molecule Inhibitor of Aurora A Kinase Using Novel *In Vivo* Pharmacodynamic Assays. *Clinical Cancer Research* **2011**, *17*, 7614–7624, doi:10.1158/1078-0432.CCR-11-1536.

12. Qi, W.; Spier, C.; Liu, X.; Agarwal, A.; Cooke, L.S.; Persky, D.O.; Chen, D.; Miller, T.P.; Mahadevan, D. Alisertib (MLN8237) an Investigational Agent Suppresses Aurora A and B Activity, Inhibits Proliferation, Promotes Endo-Reduplication and Induces Apoptosis in T-NHL Cell Lines Supporting Its Importance in PTCL Treatment. *Leuk. Res.* **2013**, *37*, 434–439, doi:10.1016/j.leukres.2012.10.017.

13. Degrassi, F.; Fiore, M.; Palitti, F. Chromosomal Aberrations and Genomic Instability Induced by Topoisomerase-Targeted Antitumour Drugs. *Current Medicinal Chemistry-Anti-Cancer Agents* **2004**, *4*, 317–325, doi:10.2174/1568011043352920.

14. Larsen, S.L.; Yde, C.W.; Laenkholm, A.-V.; Rasmussen, B.B.; Duun-Henriksen, A.K.; Bak, M.; Lykkesfeldt, A.E.; Kirkegaard, T. Aurora Kinase B Is Important for Antiestrogen Resistant Cell Growth and a Potential Biomarker for Tamoxifen Resistant Breast Cancer. *BMC Cancer* **2015**, *15*, 239, doi:10.1186/s12885-015-1210-4.

15. Clément, M.-J.; Rathinasamy, K.; Adjadj, E.; Toma, F.; Curmi, P.A.; Panda, D. Benomyl and Colchicine Synergistically Inhibit Cell Proliferation and Mitosis: Evidence of Distinct Binding Sites for These Agents in Tubulin. *Biochemistry* **2008**, *47*, 13016–13025, doi:10.1021/bi801136q.

16. Lin, H.-Y.; Li, Z.-K.; Bai, L.-F.; Baloch, S.K.; Wang, F.; Qiu, H.-Y.; Wang, X.; Qi, J.-L.; Yang, R.-W.; Wang, X.-M.; et al. Synthesis of Aryl Dihydrothiazol Acyl Shikonin Ester Derivatives as Anticancer Agents through Microtubule Stabilization. *Biochem. Pharmacol.* **2015**, *96*, 93–106, doi:10.1016/j.bcp.2015.04.021.

17. Marzaro, G.; Chilin, A. QSAR and 3D-QSAR Models in the Field of Tubulin Inhibitors as Anticancer Agents. *Curr. Top. Med. Chem.* **2014**, *14*, 2253–2262, doi:10.2174/1568026614666141130092853.

18. Marcon, F.; De Battistis, F.; Siniscalchi, E.; Crebelli, R.; Meschini, R. The Mitochondrial Poison Carbonyl Cyanide 3-Chlorophenyl Hydrazone (CCCP) Induces Aneugenic Effects in Primary Human Fibroblasts: A Possible Link between Mitochondrial Dysfunction and Chromosomal Loss. *Mutagenesis* **2022**, *37*, 155–163, doi:10.1093/mutage/geac008.

19. Brunner, M.; Albertini, S.; Würgler, F.E. Effects of 10 Known or Suspected Spindle Poisons in the *in Vitro* Porcine Brain Tubulin Assembly Assay. *Mutagenesis* **1991**, *6*, 65–70, doi:10.1093/mutage/6.1.65.

20. Nutley, E. V.; Tcheong, A.C.; Allen, J.W.; Collins, B.W.; Mo, M.; Lowe, X.R.; Bishop, J.B.; Moore, D.H.; Wyrobek, A.J. Micronuclei Induced in Round Spermatids of Mice after Stem-Cell Treatment with Chloral Hydrate: Evaluations with Centromeric DNA Probes and Kinetochore Antibodies. *Environ. Mol. Mutagen.* **1996**, *28*, 80–89, doi:10.1002/(SICI)1098-2280(1996)28:2<80::AID-EM3>3.0.CO;2-I.

21. Wei, L.; Ma, W.; Cai, H.; Peng, S.P.; Tian, H.B.; Wang, J.F.; Gao, L.; He, J.P. Inhibition of Ciliogenesis Enhances the Cellular Sensitivity to Temozolomide and Ionizing Radiation in Human Glioblastoma Cells. *Biomed Environ Sci* **2022**, *35(5)*, 419436.

22. Stavrovskaya, A.A.; Kopnin, B.P. Colcemid-induced Polyploidy and Aneuploidy in Normal and Tumour Cells *in Vitro*. *Int. J. Cancer* **1975**, *16*, 730–737, doi:10.1002/ijc.2910160505.

23. Boulos, J.C.; Saeed, M.E.M.; Chatterjee, M.; Bülbül, Y.; Crudo, F.; Marko, D.; Munder, M.; Klauck, S.M.; Efferth, T. Repurposing of the ALK Inhibitor Crizotinib for Acute Leukemia and Multiple Myeloma Cells. *Pharmaceuticals* **2021**, *14*, 1126, doi:10.3390/ph14111126.

24. Kong, Y.; Bender, A.; Yan, A. Identification of Novel Aurora Kinase A (AURKA) Inhibitors via Hierarchical Ligand-Based Virtual Screening. *J. Chem. Inf. Model.* **2018**, *58*, 36–47, doi:10.1021/acs.jcim.7b00300.

25. Meulenbeld, H.J.; Mathijssen, R.H.; Verweij, J.; de Wit, R.; de Jonge, M.J. Danusertib, an Aurora Kinase Inhibitor. *Expert Opin. Investig. Drugs* **2012**, *21*, 383–393, doi:10.1517/13543784.2012.652303.

26. Amadio, F.; Bongiorni, S.; Varalda, G.M.; Marcon, F.; Meschini, R. Di(2-Ethylexyl) Phthalate and Chromosomal Damage: Insight on Aneugenicity from the Cytochalasin-Block Micronucleus Assay. *Mutat. Res. Genet. Toxicol. Environ. Mutagen.* **2024**, *898*, 503791, doi:10.1016/j.mrgentox.2024.503791.

27. Wallin, M.; Hartley-Asp, B. Effects of Potential Aneuploidy Inducing Agents on Microtubule Assembly in Vitro. *Mutation Research - Fundamental and Molecular Mechanisms of Mutagenesis* **1993**, *287*, 17–22, doi:10.1016/0027-5107(93)90141-2.

28. Kamel, K.; Kolinski, A. Computational Study of Binding of Epothilone A to β-Tubulin. *Acta Biochim Pol* **2011**, *58(2)*, 255–260.

29. Sarsar, O.; Macar, O.; Kalefetoğlu Macar, T.; Çavuşoğlu, K.; Yalçın, E.; Acar, A. Multifaceted Investigation of Esfenvalerate-Induced Toxicity on Allium Cepa L. *Sci. Rep.* **2025**, *15*, 16977, doi:10.1038/s41598-025-01638-3.

30. Huang, S.; Lu, Y.; Li, S.; Zhou, T.; Wang, J.; Xia, J.; Zhang, X.; Zhou, Z. Key Proteins of Proteome Underlying Sperm Malformation of Rats Exposed to Low Fenvalerate Doses Are Highly Related to <scp>P53</Scp>. *Environ. Toxicol.* **2021**, *36*, 1181–1194, doi:10.1002/tox.23117.

31. Yadav, M.; Dhagat, S.; Eswari, J.S. Structure Based Drug Design and Molecular Docking Studies of Anticancer Molecules Paclitaxel, Etoposide and Topotecan Using Novel Ligands. *Curr. Drug Discov. Technol.* **2020**, *17*, 183–190, doi:10.2174/1570163816666190307102033.

32. Ardalani, H.; Avan, A.; Ghayour-Mobarhan, M. Podophyllotoxin: A Novel Potential Natural Anticancer Agent. *Avicenna J Phytomed* **2017**, *7(4)*, 285–294.

33. Nezami, R.; Otis, C.; Boyer, A.; Blanchard, J.; Moreau, M.; Pelletier, J.-P.; Martel-Pelletier, J.; Godoy, P.; Troncy, E. Surveillance of Ancylostoma Caninum in Naturally Infected Dogs in Quebec, Canada, and Assessment of Benzimidazole Anthelmintics Reveal a Variable Efficacy with the Presence of a Resistant Isolate in Imported Dogs. *Vet. Parasitol. Reg. Stud. Reports* **2024**, *52*, 101036, doi:10.1016/j.vprsr.2024.101036.

34. Lacey, E.; Prichard, R.K. Interactions of Benzimidazoles (BZ) with Tubulin from BZ-Sensitive and BZ-Resistant Isolates of Haemonchus Contortus. *Mol. Biochem. Parasitol.* **1986**, *19*, 171–181, doi:10.1016/0166-6851(86)90122-2.

35. Rathinasamy, K.; Jindal, B.; Asthana, J.; Singh, P.; Balaji, P. V; Panda, D. Griseofulvin Stabilizes Microtubule Dynamics, Activates P53 and Inhibits the Proliferation of MCF-7 Cells Synergistically with Vinblastine. *BMC Cancer* **2010**, *10*, 213, doi:10.1186/1471-2407-10-213.

36. Das, S.; Paul, S. Exploring the Binding Sites and Binding Mechanism for Hydrotrope Encapsulated Griseofulvin Drug on γ-Tubulin Protein. *PLoS One* **2018**, *13*, e0190209, doi:10.1371/journal.pone.0190209.

37. Zacharaki, P.; Stephanou, G.; Demopoulos, N.A. Comparison of the Aneugenic Properties of Nocodazole, Paclitaxel and Griseofulvin *in Vitro* . Centrosome Defects and Alterations in Protein Expression Profiles. *Journal of Applied Toxicology* **2013**, *33*, 869–879, doi:10.1002/jat.2745.

38. Hauf, S.; Cole, R.W.; LaTerra, S.; Zimmer, C.; Schnapp, G.; Walter, R.; Heckel, A.; van Meel, J.; Rieder, C.L.; Peters, J.-M. The Small Molecule Hesperadin Reveals a Role for Aurora B in Correcting Kinetochore–Microtubule Attachment and in Maintaining the Spindle Assembly Checkpoint. *J. Cell Biol.* **2003**, *161*, 281–294, doi:10.1083/jcb.200208092.

39. Lopus, M.; Smiyun, G.; Miller, H.; Oroudjev, E.; Wilson, L.; Jordan, M.A. Mechanism of Action of Ixabepilone and Its Interactions with the ΒIII-Tubulin Isotype. *Cancer Chemother. Pharmacol.* **2015**, *76*, 1013–1024, doi:10.1007/s00280-015-2863-z.

40. Churchill, C.D.M.; Klobukowski, M.; Tuszynski, J.A. The Unique Binding Mode of Laulimalide to Two Tubulin Protofilaments. *Chem. Biol. Drug Des.* **2015**, *86*, 190–199, doi:10.1111/cbdd.12475.

41. Gajewski, M.M.; Alisaraie, L.; Tuszynski, J.A. Peloruside, Laulimalide, and Noscapine Interactions with Beta-Tubulin. *Pharm. Res.* **2012**, *29*, 2985–2993, doi:10.1007/s11095-012-0809-2.

42. Efthimiou, M.; Andrianopoulos, C.; Stephanou, G.; Demopoulos, N.A.; Nikolaropoulos, S.S. Aneugenic Potential of the Nitrogen Mustard Analogues Melphalan, Chlorambucil and p-N,N-Bis(2-Chloroethyl)Aminophenylacetic Acid in Cell Cultures in Vitro. *Mutation Research - Fundamental and Molecular Mechanisms of Mutagenesis* **2007**, *617*, 125–137, doi:10.1016/j.mrfmmm.2007.01.009.

43. Acharya, B.R.; Choudhury, D.; Das, A.; Chakrabarti, G. Vitamin K3 Disrupts the Microtubule Networks by Binding to Tubulin: A Novel Mechanism of Its Antiproliferative Activity. *Biochemistry* **2009**, *48*, 6963–6974, doi:10.1021/bi900152k.

44. Kallio, M.; Lähdetie, J. Fragmentation of Centromeric DNA and Prevention of Homologous Chromosome Separation in Male Mouse Meiosis *in Vivo* by the Topoisomerase II Inhibitor Etoposide. *Mutagenesis* **1996**, *11*, 435–443, doi:10.1093/mutage/11.5.435.

45. Kallio, M.; Lähdetie, J. Effects of the DNA Topoisomerase II Inhibitor Merbarone in Male Mouse Meiotic Divisions in Vivo: Cell Cycle Arrest and Induction of Aneuploidy. *Environ. Mol. Mutagen.* **1997**, *29*, 16–27, doi:10.1002/(SICI)1098-2280(1997)29:1<16::AID-EM3>3.0.CO;2-B.

46. Molino, C.; Filippi, S.; Giovani, G.; Caccia, A.; Meschini, R.; Angeletti, D. Effects of Phthalates on Marine Organisms: Cytotoxicity and Genotoxicity of Mono-(2-Ethylhexyl)-Phthalate (MEHP) on European Sea Bass (Dicentrarchus Labrax) Embryonic Cell Line. *Ann. Ist. Super. Sanita* **2023**, *59*, 68–75, doi:10.4415/ANN_23_01_10.

47. Bush, T.L.; Payton, M.; Heller, S.; Chung, G.; Hanestad, K.; Rottman, J.B.; Loberg, R.; Friberg, G.; Kendall, R.L.; Saffran, D.; et al. AMG 900, a Small-Molecule Inhibitor of Aurora Kinases, Potentiates the Activity of Microtubule-Targeting Agents in Human Metastatic Breast Cancer Models. *Mol. Cancer Ther.* **2013**, *12*, 2356–2366, doi:10.1158/1535-7163.MCT-12-1178.

48. Payton, M.; Bush, T.L.; Chung, G.; Ziegler, B.; Eden, P.; McElroy, P.; Ross, S.; Cee, V.J.; Deak, H.L.; Hodous, B.L.; et al. Preclinical Evaluation of AMG 900, a Novel Potent and Highly Selective Pan-Aurora Kinase Inhibitor with Activity in Taxane-Resistant Tumor Cell Lines. *Cancer Res.* **2010**, *70*, 9846–9854, doi:10.1158/0008-5472.CAN-10-3001.

49. Lamberti, A.; Sanges, C.; Longo, O.; Chambery, A.; Maro, A.; Parente, A.; Masullo, M.; Arcari, P. Analysis of Nickel-Binding Peptides in a Human Hepidermoid Cancer Cell Line by Ni-NTA Affinity Chromatography and Mass Spectrometry. *Protein Pept. Lett.* **2008**, *15*, 1126–1131, doi:10.2174/092986608786071157.

50. Guo, D.; Ma, J.; Su, W.; Xie, B.; Guo, C. Contribution of Reactive Oxygen Species (ROS) to Genotoxicity of Nitrobenzene on V. Faba. *Ecotoxicology* **2014**, *23*, 657–664, doi:10.1007/s10646-014-1230-x.

51. Bolt, H.M.; Bonacker, D.; Stoiber, T.; B�hm, K.J.; Unger, E.; Degen, G.H.; Thier, R. Chromosomal Genotoxicity of Nitrobenzene and Benzonitrile. *Arch. Toxicol.* **2004**, *78*, 49–57, doi:10.1007/s00204-003-0508-1.

52. Guzmán-Ocampo, D.C.; Aguayo-Ortiz, R.; Cano-González, L.; Castillo, R.; Hernández-Campos, A.; Dominguez, L. Effects of the Protonation State of Titratable Residues and the Presence of Water Molecules on Nocodazole Binding to Β-Tubulin. *ChemMedChem* **2018**, *13*, 20–24, doi:10.1002/cmdc.201700530.

53. Samson; Donoso; Heller-Bettinger; Watson; Himes Nocodazole Action on Tubulin Assembly, Axonal Ultrastructure and Fast Axoplasmic Transport. *J Pharmacol Exp Ther* **1979**, *208(3)*, 411–417.

54. Chen, Y.; Wen, C.; Zhong, S.; Huang, L.; Xiang, Y.; Ou, Y.; Li, L.; Tang, W.; Zhou, C.; Wu, Z.; et al. Oxfendazole Induces Apoptosis in Ovarian Cancer Cells by Activating JNK/MAPK Pathway and Inducing Reactive Oxygen Species Generation. *Biol. Pharm. Bull.* **2023**, *46*, b23-00349, doi:10.1248/bpb.b23-00349.

55. Heard, P.L.; Rubitski, E.E.; Spellman, R.A.; Schuler, M.J. Phenolphthalein Induces Centrosome Amplification and Tubulin Depolymerization *in Vitro*. *Environ. Mol. Mutagen.* **2013**, *54*, 308–316, doi:10.1002/em.21781.

56. Armstrong, M.J.; Gara, J.P.; Gealy III, R.; Greenwood, S.K.; Hilliard, C.A.; Laws, G.M.; Galloway, S.M. Induction of Chromosome Aberrations in Vitro by Phenolphthalein: Mechanistic Studies. *Mutation Research - Fundamental and Molecular Mechanisms of Mutagenesis* **2000**, *457*, 15–30, doi:10.1016/S0027-5107(00)00119-6.

57. Desbene, S.; Giorgi-Renault, S. Drugs That Inhibit Tubulin Polymerization: The Particular Case of Podophyllotoxin and Analogues. *Current Medicinal Chemistry-Anti-Cancer Agents* **2012**, *2*, 71–90, doi:10.2174/1568011023354353.

58. Sackett, D.L. Podophyllotoxin, Steganacin and Combretastatin: Natural Products That Bind at the Colchicine Site of Tubulin. *Pharmacol. Ther.* **1993**, *59*, 163–228, doi:10.1016/0163-7258(93)90044-E.

59. Cortese, B.W. Podophyllotoxin as a Probe for the Colchicine Binding Site of Tubulin. *J Biol Chem* **1977**, 1134–1340.

60. Srivastava, P.; Panda, D. Rotenone Inhibits Mammalian Cell Proliferation by Inhibiting Microtubule Assembly through Tubulin Binding. *FEBS J.* **2007**, *274*, 4788–4801, doi:10.1111/j.1742-4658.2007.06004.x.

61. Diaz-Corrales, F.J.; Asanuma, M.; Miyazaki, I.; Miyoshi, K.; Ogawa, N. Rotenone Induces Aggregation of γ-Tubulin Protein and Subsequent Disorganization of the Centrosome: Relevance to Formation of Inclusion Bodies and Neurodegeneration. *Neuroscience* **2005**, *133*, 117–135, doi:10.1016/j.neuroscience.2005.01.044.

62. Arnst, J. When Taxol Met Tubulin. *Journal of Biological Chemistry* **2020**, *295*, 13994–13995, doi:10.1074/jbc.CL120.015923.

63. Han, Y.; Chaudhary, A.G.; Chordia, M.D.; Sackett, D.L.; Perez-Ramirez, B.; Kingston, D.G.I.; Bane, S. Interaction of a Fluorescent Derivative of Paclitaxel (Taxol) [1] with Microtubules and Tubulin−Colchicine. *Biochemistry* **1996**, *35*, 14173–14183, doi:10.1021/bi960774l.

64. Ashrafuzzaman, M.; Tseng, C.; Duszyk, M.; Tuszynski, J.A. Chemotherapy Drugs Form Ion Pores in Membranes Due to Physical Interactions with Lipids. *Chem. Biol. Drug Des.* **2012**, *80*, 992–1002, doi:10.1111/cbdd.12060.

65. Grover, S.; Boyé, O.; Getahun, Z.; Brossi, A.; Hamel, E. Chloroacetates of 2- and 3-Demethylthiocolchicine: Specific Covalent Interactions with Tubulin with Preferential Labeling of the β-Subunit. *Biochem. Biophys. Res. Commun.* **1992**, *187*, 1350–1358, doi:10.1016/0006-291X(92)90451-P.

66. Ke, D.; Meng, H.; Lei, W.; Zheng, Y.; Li, L.; Wang, M.; Zhong, R.; Wang, M.; Chen, F. Prevalence of H6Y Mutation in β-Tubulin Causing Thiophanate-Methyl Resistant in Monilinia Fructicola from Fujian, China. *Pestic. Biochem. Physiol.* **2022**, *188*, 105262, doi:10.1016/j.pestbp.2022.105262.

67. Gizatullin, F.; Yao, Y.; Kung, V.; Harding, M.W.; Loda, M.; Shapiro, G.I. The Aurora Kinase Inhibitor VX-680 Induces Endoreduplication and Apoptosis Preferentially in Cells with Compromised P53-Dependent Postmitotic Checkpoint Function. *Cancer Res.* **2006**, *66*, 7668–7677, doi:10.1158/0008-5472.CAN-05-3353.

68. Harrington, E.A.; Bebbington, D.; Moore, J.; Rasmussen, R.K.; Ajose-Adeogun, A.O.; Nakayama, T.; Graham, J.A.; Demur, C.; Hercend, T.; Diu-Hercend, A.; et al. VX-680, a Potent and Selective Small-Molecule Inhibitor of the Aurora Kinases, Suppresses Tumor Growth in Vivo. *Nat. Med.* **2004**, *10*, 262–267, doi:10.1038/nm1003.

69. Shi, Q.; Yang, H.; Chen, Y.; Zheng, N.; Li, X.; Wang, X.; Ding, W.; Zhang, B. Developmental Neurotoxicity of Trichlorfon in Zebrafish Larvae. *Int. J. Mol. Sci.* **2023**, *24*, 11099, doi:10.3390/ijms241311099.

70. Doherty, A.T.; Ellard, S.; Parry, E.M.; Parry, J.M. A Study of the Aneugenic Activity of Trichlorfon Detected by Centromere-Specific Probes in Human Lymphoblastoid Cell Lines. *Mutation Research - Fundamental and Molecular Mechanisms of Mutagenesis* **1996**, *372*, 221–231, doi:10.1016/S0027-5107(96)00142-X.

71. ROBINSON, M.W.; TRUDGETT, A.; HOEY, E.M.; FAIRWEATHER, I. Triclabendazole-Resistant *Fasciola Hepatica* : β-Tubulin and Response to *in Vitro* Treatment with Triclabendazole. *Parasitology* **2002**, *124*, 325–338, doi:10.1017/S003118200100124X.

72. Olivares-Ferretti, P.; Beltrán, J.F.; Salazar, L.A.; Fonseca-Salamanca, F. Protein Modelling and Molecular Docking Analysis of Fasciola Hepatica β-Tubulin's Interaction Sites, with Triclabendazole, Triclabendazole Sulphoxide and Triclabendazole Sulphone. *Acta Parasitol.* **2023**, *68*, 535–547, doi:10.1007/s11686-023-00692-z.

73. Weng, H.; Li, J.; Zhu, H.; Carver Wong, K.F.; Zhu, Z.; Xu, J. An Update on the Recent Advances and Discovery of Novel Tubulin Colchicine Binding Inhibitors. *Future Med. Chem.* **2023**, *15*, 73–95, doi:10.4155/fmc-2022-0212.

74. Jordan, A.; Hadfield, J.A.; Lawrence, N.J.; McGown, A.T. Tubulin as a Target for Anticancer Drugs: Agents Which Interact with the Mitotic Spindle. *Med. Res. Rev.* **1998**, *18*, 259–296, doi:10.1002/(SICI)1098-1128(199807)18:4<259::AID-MED3>3.0.CO;2-U.

75. Bayssas, M.; Gouveia, J.; de Vassal, F.; Misset, J.-L.; Schwarzenberg, L.; Ribaud, P.; Musset, M.; Jasmin, C.; Hayat, M.; Mathé, G. Vindesine: A New Vinca Alkaloid. In; 1980; pp. 91–97.

76. Kruczynski, A.; Barret, J.-M.; Etiévant, C.; Colpaert, F.; Fahy, J.; Hill, B.T. Antimitotic and Tubulin-Interacting Properties of Vinflunine, a Novel Fluorinated Vinca Alkaloid. *Biochem. Pharmacol.* **1998**, *55*, 635–648, doi:10.1016/S0006-2952(97)00505-4.

77. Fabre, C.; Czaplicki, J.; Wright, M.; Hill, B.; Barret, J.M.; Fahy, J.; Milon, A. Differential Binding to the α/β-Tubulin Dimer of Vinorelbine and Vinflunine Revealed by Nuclear Magnetic Resonance Analyses. *Biochem. Pharmacol.* **2002**, *64*, 733–740, doi:10.1016/S0006-2952(02)01255-8.

78. Helmke, C.; Becker, S.; Strebhardt, K. The Role of Plk3 in Oncogenesis. *Oncogene* **2016**, *35*, 135–147, doi:10.1038/onc.2015.105.

79. Rudolph, D.; Impagnatiello, M.A.; Blaukopf, C.; Sommer, C.; Gerlich, D.W.; Roth, M.; Tontsch-Grunt, U.; Wernitznig, A.; Savarese, F.; Hofmann, M.H.; et al. Efficacy and Mechanism of Action of Volasertib, a Potent and Selective Inhibitor of Polo-Like Kinases, in Preclinical Models of Acute Myeloid Leukemia. *J. Pharmacol. Exp. Ther.* **2015**, *352*, 579–589, doi:10.1124/jpet.114.221150.
